# Unmasking the Hidden Culprit: A Coma Mimicry in a Child Bitten by Cobra

**DOI:** 10.1155/2024/6630842

**Published:** 2024-01-09

**Authors:** Aphirak Mekmangkonthong, Khanittha Khusiwilai, Sudathip Paticheep, Duangtip Tiacharoen

**Affiliations:** ^1^Thammasat University Hospital, Khlong Nueng, Thailand; ^2^Neurology Unit, Department of Pediatrics, Faculty of Medicine, Thammasat University, Bangkok, Thailand; ^3^Critical Care Unit, Department of Pediatrics, Faculty of Medicine, Thammasat University, Bangkok, Thailand

## Abstract

Snake bite is a significant public health concern, particularly in tropical regions. Individuals who are bitten by neurotoxin snake commonly present with ptosis, ophthalmoparesis, muscle weakness, and diminished or absent of deep tendon reflexes. However, accurately determining the occurrence of a snakebite can sometimes be challenging, potentially leading to misdiagnosis. We present the case of a 2-year-old boy with sudden cardiac arrest. Following a brief resuscitation, he had return of spontaneous circulation. Despite normal electroencephalography results, the patient continued to have absence of brainstem reflexes, spontaneous breathing, and movement. Cobra antivenom was promptly initiated based on suspicions of a neurotoxic snakebite, resulting in a rapid recovery of the patient's condition. We proposed that neurotoxin snake envenomation should be considered in patients with sudden cardiac arrest with uncertain cause, particularly in snake endemic areas.

## 1. Introduction

Snakebite envenomation has emerged as a significant global issue, recognized by World Health Organization (WHO) as a “neglected tropical disease” [1, 2]. Snake bite deaths are most prevalent in south Asia, southeast Asia, and sub-Saharan Africa [3]. In Thailand, an average of 6,496 snakebite incidents have occurred annually over the past two decades, with a recent incident of 12.07 patients per 100,000 people [4]. The monocled cobra and the Malayan krait are the primary venomous neurotoxic snakes responsible for fatalities [5]. Common presentations of neurotoxin envenomation include muscle weakness, ophthalmoparesis, and ptosis. In severe instances, patients may progress to respiratory failure as a result of compromised respiratory muscle function and potentially death. However, in uncommon scenarios, the absence of brainstem reflexes can be caused by postsynaptic blockage of cranial nerves [1, 2, 6]. This particular circumstance often perplexes physicians, who may mistakenly diagnose brain death, unaware of the underlying correctable cause.

## 2. Case Report

The patient was a previously healthy, 2-year-old boy, who was referred to our hospital following an unknown cause of sudden cardiac arrest. Thirty minutes prior to the cardiovascular arrest, his right foot was trapped between floor gaps while he was playing at his home, leading to its submersion in sewage. He cried and ran back to his mother, who brought him to the first hospital. While the patient's vital signs were being assessed, he suddenly developed air hunger. Oxygen saturation was unmeasurable. He suddenly collapsed and became pulseless, necessitating immediate cardiopulmonary resuscitation (CPR) and intubation. No medication was administered during CPR. He had return of spontaneous circulation, and vital signs were stabilized after 2 minutes of resuscitation.

**Figure 1:**
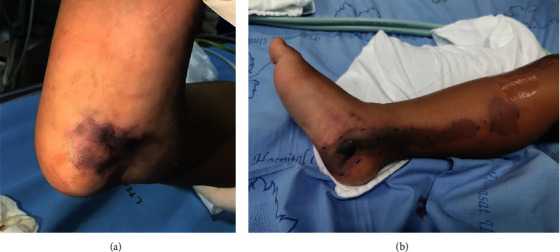
Discoloration was observed on the right medial ankle upon the patient's admission (a). Subsequently, on the third day of admission, a follow-up examination revealed the persistence of discoloration at the same location (b).

Physical examination revealed a comatose state. His vital signs that included blood pressure and heart rate fluctuated. There was no spontaneous breathing. Pupils were 3 mm in diameter, fixed. The oculocephalic reflex (doll's eyes) and gag's reflex were absent. He had severe hypotonia, no muscle contraction after noxious stimuli, and absent of all deep tendon reflexes. Plantar responses and clonus were absent. Stiffness of neck was absent. Notably, a bruise was noted at the medial right malleolus; however, there were no discernible fang marks present in the area (Figure 1(a)).

**Figure 2:**
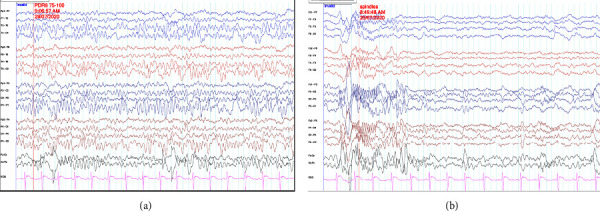
On the second day of admission, video electroencephalography (EEG) monitoring was conducted. The patient's clinical presentation was consistent with a diagnosis of the comatose stage. The recorded EEG findings revealed normal patterns during wakefulness (a) and normal nonrapid eye movement (non-REM) sleep stage 2 (b).

**Figure 3:**
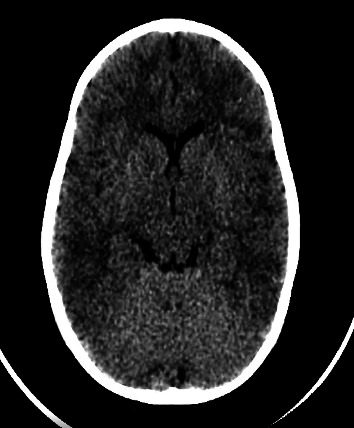
Computed tomography of the brain revealed diffused brain edema with white cerebellum sign.

The laboratory results including complete blood count, serum electrolytes, and coagulogram were within the normal limits. Venous clotting time was 8 minutes (<20 minutes). Electrocardiography showed no abnormal heart rhythms, while the echocardiogram detected no structural heart abnormalities. Electroencephalography examinations unveiled unremarkable patterns during both wakefulness (Figure 2(a)) and sleep (Figure 2(b)) states. Notably, computed tomography scans with contrast media of the brain revealed generalized brain edema and white cerebellum sign (Figure 3).

Two days after admission, his neurological status remained in comatose state. There were no observable brainstem reflexes, voluntary movement, or spontaneous breathing. The bruise on his right ankle progressively extended below the knee, exhibiting necrotic changes in the skin tissues (Figure 1(b)). Upon realizing that cobras were frequently located in the patient's suburban home and the skin necrosis had similarities to a cobra bite, suspicions arose about the possibility of snake envenomation as a potential underlying cause. Consequently, the administration of Thai cobra antivenom was initiated. Within twenty minutes of receiving the antivenom, the patient began exhibiting minimal movements of the eyes and limbs, originating from the distal extremities. He was extubated 1 day after administration of antivenom. Over the course of several weeks of rehabilitation, the patient gradually recovered without experiencing any lasting neurological impairments. A positive result was obtained from the Enzyme-Linked Immunosorbent Assay (ELISA) technique utilized for detecting cobra venom toxins in the patient's serum. This unequivocally confirms the diagnosis of cobra envenomation, which induced a cardiac arrest and resulted in the absence of brainstem reflexes, spontaneous movements, and respiration, thereby mimicking a state of brain death.

## 3. Discussion

Sudden cardiac arrest of unknown etiology presents physicians with diagnostic challenges and decision-making dilemmas [1]. In such cases, it is crucial to prioritize the identification and correction of reversible and treatable causes of cardiac arrest. However, in patients with neurotoxin snake envenomation, rare presentations of absence of the brainstem functions and spontaneous respiration may lead to the diagnosis of comatose stage or even brain dead. Consequently, the pursuit of appropriate treatment is delayed, potentially redirecting the patient towards palliative care.

Muscle paralysis following a cobra bite results from the postsynaptic blockade of neuromuscular junction receptors. This effect extends beyond the truncal and limb muscles, affecting the respiratory muscles as well [2]. As a consequence, respiration becomes rapidly compromised, leading to hypoxia and, ultimately, cardiac arrest. The absence of discernible fang marks further compounds the diagnostic challenge in our patient, adding an additional layer of complexity to the assessment process.

In addition to physical examination, the utilization of electroencephalography (EEG) assumes a crucial role in differentiating causes of clinically severe encephalopathy following a brief cardiac arrest episode in our patient. Despite the unresponsiveness of the patient and the absence of brainstem reflexes, the presence of normal awake and sleep EEG patterns did not align with the observed clinical presentation. Regarding the electroencephalographic features associated with snake bites, a study by Ramachandran et al. reported various EEG patterns in patients, ranging from normal, similar to our patient, to the presence of diffuse slow delta waves [7]. The conjunction of atypical absence of deep tendon reflexes and the progression of necrotic skin lesions led us to suspect a cobra bite. Consequently, administration of the Thai Red Cross cobra antivenom was initiated, resulting in a rapid neurological recovery in the patient. Consistent with prior literature, the response to antivenom therapy was striking [6, 8, 9]. Thus, clinicians should consider neurotoxin snake bites as one of the potential diagnoses in patients who present with sudden-onset coma without an obvious etiology.

## 4. Conclusion

We report a 2-year-old boy who was bitten by cobra and presented with sudden cardiac arrest, followed by comatose state. This case report underscores the significance of considering a venomous snake bite as a potential diagnostic suspicion in patients with sudden cardiac arrest, in comatose state, and absence of brainstem reflexes, even absence of fang marks. Furthermore, the report emphasizes the importance of utilizing electroencephalogram (EEG) in evaluating comatose patients, especially when the EEG findings do not align with the patient's clinical presentation.

## Data Availability

No data were used in this study.
